# Qualitative Approach to Environmental Risk Assessment in Transport

**DOI:** 10.3390/ijerph17155494

**Published:** 2020-07-30

**Authors:** Zdenek Dvorak, David Rehak, Andrej David, Zoran Cekerevac

**Affiliations:** 1 Faculty of Security Engineering, University of Zilina, 01026 Zilina, Slovakia; 2 Faculty of Safety Engineering, VSB—Technical University of Ostrava, 70030 Ostrava, Czech Republic; david.rehak@vsb.cz; 3 Faculty of Operation and Economics of Transport and Communications, University of Zilina, 01026 Zilina, Slovakia; andrej.david@fpedas.uniza.sk; 4 Faculty of Business and Law, Union—Nikola Tesla University, 11000 Belgrade, Serbia; zoran@cekerevac.eu

**Keywords:** risk assessment, transport sector, environment, common safety method, decision support system, expert evaluation

## Abstract

The purpose of this paper is to present the development of a qualitative approach to environmental risk assessment (QAERA) in transport. The approach is described as a model developed for the future software tool which will be utilizable as a risk decision support system. The basic part is aimed on developing a quantitative environmental risk assessment. Thus, this paper describes a set of 6 pillars of safety and security. Accordingly, the paper contains both chosen safety and security indicators and selected criteria for assessing the risk of launching the environmental change of global model thinking in the transport sector. The environmental risk assessment as a global model of thinking was originally based on historical experience but, nowadays, it is changing. Based on new expert knowledge, more precisely, on input of new global data, paper displays an environmental risk assessment with actual interpretation. The discussion of the paper is oriented to support research results, a new knowledge-oriented approach to global climate changes, using suitable risk assessment methods and technics. The result of the paper is a new approach for the modeling of environmental risk assessment in the transport sector.

## 1. Introduction

Transport, as one of the most important sectors of economics, has been developing gradually. Development of mankind is linked to the development of transport systems. The history of transport is mainly associated with the invention of the wheel and the development of inland waterway transport. In addition, maritime transport has played a key role in discovering new continents. With the development of the industrialization of society, railway transport began to develop significantly hand in hand with the discovery of a steam engine. The discovery of an internal combustion and diesel engine led to the development of road transport. The latest results of science and research have been fundamental elements at gradual development of each mode of transport. For many decades, the impact of transport on the environment has not been fundamentally addressed. To be specific, the first research did not take place until the middle of the twentieth century. Globally, the number of vehicles is increasing from year to year, and although the technical parameters of internal combustion engines are improving, global emissions are growing.

Emphasizing the environmental aspect of individual modes of transport should be an important aspect of the transport policy of an advanced society. The European Commission regularly issues recommendations and directives that address some areas of transport development. Individual states should define the priorities of transport development in their transport policy, mainly for ensuring transport services, but also for expected environmental impacts. Essentially, the authors of the article come from central and eastern Europe. In conjunction with this, the examples of the transport policy of the Czech Republic, Slovakia, and Serbia show that these countries have a remarkably similar transport policy, where the aspect of environmental protection plays a less important role.

The traditional scientific research plan has historically been focused on technical and technological processes. The challenge today is security with its multi-level and multi-union complexity. If safety research is understood in the above concept as a multi-level and multi-sectoral problem, then the challenge of addressing the issue of the environmental risk assessment will be met. The methodology will become a key element for setting appropriate indicators that will take into account not only the economic benefits for the company but will also address the societal impact in the social, health, and environmental fields. Drawing on this, simplifying efficiency and risk assessments have now led to absurd shipments of raw materials and semi-finished products in globalized companies, regardless of the full cost.

Current methods of environmental risk assessment are mainly of a quantitative or hybrid nature. In engineering, these methods are most commonly used in industry, where they account for more than 50% of existing risk assessment methods [1]. In the field of transport, these methods are used mainly in the context of the assessment of mobile risk sources [2,3]. However, these methods require a large amount of specific data, which is very time consuming to collect [4]. For this reason, it is convenient to use at first some of the appropriate qualitative methods for the preliminary risk analysis [5,6]. These methods are time-saving but, on the contrary, also quite general.

Referring to the above, it can be stated that even today there is no suitable available method dealing with the qualitative assessment of environmental risks arising from normal traffic. Based on this, the paper aims to present a newly proposed qualitative approach to environmental risk assessment in the transport sector. The essence of this approach is a preliminary environmental risk assessment, which is based on the application of selected safety and security indicators and selected risk assessment criteria in the implementation of environmental change in the global model of thinking in the transport sector.

## 2. Literature Review

The preparation of a paper focusing on environmental risk assessment in transport requires a broader framework of solutions. Therefore, we primarily examined information sources that play a decisive role in the field of environmental protection. Subsequently, attention was paid to the areas of sustainable development, transport, safety and security.

### 2.1. Environmental Area

The first paper to be discussed is the paper Biodiversity Loss and its Impact on Humanity, written by a broad team of authors led by Cardinale et al. [7]. The paper has focused on research since 1980, evaluating the last 40 years of life on Earth. Gradually, significant climate changes are taking place, which are accompanied by extreme weather events and often lead to the extinction of some animal and plant species. Entire ecosystems disappear irretrievably. As mentioned in the paper, in 2005, the Millennium Ecosystem Assessment was published for the first time. The condition and trends in the world’s ecosystems, and the services they provide, highlighted two distinct foci of biodiversity and ecosystem functioning (BEF) and biodiversity and ecosystem services (BES) research. Selecting from the published Consensus statements, there is currently clear evidence that biodiversity loss reduces the efficiency of ecological communities that capture biologically important resources, produce biomass, decompose, and recycle biologically important nutrients. Furthermore, there is evidence that greater biodiversity increases the stability of ecosystem functions over time. Moreover, four emerging trends were defined in the research. In our research on environmental risk assessment, we found that diversity effects grow stronger with time, and may increase at larger spatial scales. In BES research—for example, if we want to know how biodiversity affects the ability of ecosystems to remove carbon dioxide from the atmosphere and store carbon in the long term, we need to consider the net impact of biodiversity on photosynthesis (exchange of carbon dioxide for oxygen).

Changes in the environment have taken place continuously in the history of the planet Earth. The research is focused on different parts of the environment. An example to be used in our publication is the article 2500-Year Climate and Environmental Record Inferred from Subfossil Chironomids from Lugu Lake, by the author team led by Chang et al. [8] from 2018. The research focused on multi-proxy indicators for catchment erosion at Lugu Lake puts forward a methodological approach that the issue of environmental change must be considered in a long time series.

Another relevant source is Connor et al. [9] Long-Term Population Dynamics: Theory and Reality in a Peatland Ecosystem, which is focused on the research of environmental change in a selected region. Jimenez and Flores [10] focused on reducing carbon dioxide emissions in large agglomerations, where transport plays a big role. Kondratyev et al. [11] was involved in research of global carbon cycle and climate change. Similarly, Koblen et al. [12] focused on reducing emissions from aviation transport. This research was followed by Koscak et al. [13] which dealt with negative environmental impact of winter airport maintenance.

Within the European Universities Association, there was an established waterborne technology platform, which integrates research activities within maritime transport. The platform has defined seven key trends for maritime transport, which touch on the topic addressed and will be addressed in the paper [14].

### 2.2. Sustainable Development Area

As a part of global solutions, world conferences on environmental protection were organized in the past. Gradually, this agenda became one of the 17 goals of the transforming world called the 2030 Agenda for Sustainable Development. This agenda was approved by the United Nations and it contains 197 targets in addition to 17 basic goals. Some of them, directly and indirectly, affect the issues of the environment and sustainable transport [15]. Jakobsen [16] subsequently brought a new perspective on ecological economics.

As for these goals and targets, four goals are relevant to our research: Goal 4—Ensure lifelong learning opportunities; Goal 9—Built resilient infrastructure, promote inclusive and sustainable industrialization and foster innovation; Goal 11—Make city and human settlements inclusive safe, resilient, and sustainable; and Goal 13—Take urgent action to combat climate change and its impacts. In the next part, attention will be paid to them [15].

### 2.3. Transport Area

**Figure 1:**
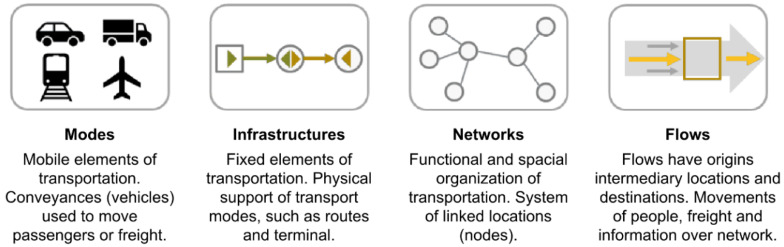
Components of transport modes [17].

A lot of research teams around the world are involved in assessing environmental change and its impact on transport and transport infrastructure. Extreme weather events, seismic and geological changes occur in all regions of the world. However, the first limitation of our research is the focus on the central European environment, as examples will be events from the Czech Republic, Slovakia, and Serbia. All three countries have remarkably similar relief. On the whole, there are mountain ranges with transport infrastructure and lowlands with large rivers. The nature of the threats to transport and transport infrastructure is similar. For this reason, the writers focus their research on four basic key components—Modes, Infrastructures, Networks, and Flows (see Figure 1).

As a significant contribution to the field of environmental risk assessment in transport, we present the RAIN project (i.e. Risk Analysis of Infrastructure Networks) solved in the years 2014–2016. The project pointed up the impact of weather extremes on transport and energy. The project resulted in building up a new framework for assessing the risks posed by weather extremes—floods, lightning, landslides, extreme droughts, frosts, and storms [18].

The theoretical framework of risk assessment and information support in railway and road transport was also addressed in the publications Assessment of Critical Infrastructure Elements in Transport [19], Crisis Management Decision Support System in Railway Infrastructure Company [20], and Multi-agent System for Decreasing of Risk in Road Transport [21].

Dzunda et al. [22] dealt with the synthesis of selected aspects of navigation systems to increase air safety. Havel et al. [23] focused their attention on a new route intersection-minimum distance model. Sousek and Dvorak [24] researched the transport of dangerous shipments. Fuchs et al. [25], within the framework of the BIOTRA project and a series of publications, concentrated on the transport of dangerous goods and the assessment of its possible impacts on the environment in the vicinity of roads (line objects). Hoterova and Dvorak [26] discussed comparing CO_2_ emissions in rail and road transport.

The Water Transport Technology Platform welcomes the Council Conclusions entitled “EU Water Transport Sector—Looking to the future: Towards a carbon-neutral, zero-accident, automated and competitive EU water transport sector”, endorsed by the Council on 5 June 2020. These conclusions are politically very relevant as they clearly demonstrate the views of the Member States on European policy on the future of waterborne transport. The conclusions underline this strategy—the growing importance of the European waterway sector and the need for enhanced research, development, and innovation to achieve the objectives set out in the Council conclusions. Key trends influencing the maritime sector are, e.g., climate change, continuous population growth and urbanization, food and water demand, increasing expectations for health, safety and security and, last but not least, developing countries. On the whole, these constituent factors will increase their share in global economic growth, increase in energy consumption, and fast development of information and communication technologies [27].

### 2.4. Safety and Security Area

Transport, as a specific area of economic activity, and the provision of transport services for people requires the continuous improvement of all activities and subsystems. During the evaluating of traffic, people focus on attributes such as time, price, quality, safety, and environmental impact. As a part of the evaluation of safety and security aspects, researchers write of the point, line, and area objects. These attributes and aspects were defined within the projects RAIN [18], OKISD (i.e. Critical Infrastructure Protection in Sector Transportation in the Slovak language) [28], and RESILIENCE 2015 [29].

The risk assessment for the environmental area was addressed in the following publications: Dobes et al. [30], Approach of the Czech Republic to the Prevention of Environmentally Oriented Terrorism. Kelemen and Jevcak [31] addressed the specifics of security management in aviation. Kirschenstein et al. [32] discussed research into storms and the resulting threats to air traffic. Polishchuk et al. [33], in their research, dealt with a fuzzy model of risk assessment for environmental for start-up projects in the air transporter sector. Rehak et al. [34] spoke of Safety and Security Issues in Technical Infrastructures.

The decade since 2010 has been aimed at significantly reducing CO_2_ production by car and aircraft engines. Because of that, many studies were carried out to compare the production of emissions in different modes of transport. The effects of extreme weather events pose a visible response of the Earth to increasing global greenhouse gas emissions. Gradual climate change is mainly reflected in the ever-increasing global temperature, which is leading to the gradual melting of glaciers around the North and South Pole. The significant reduction in the world’s glaciers is also the result of the global deforestation and forest systems. Currently, 40% of the land on Earth is covered by deserts, and their size is growing from year to year. First, according to historical meteorological measurements over the last hundred years, seven of the ten warmest years occurred between 2000 and 2019. Second, according to research by the European Severe Storms Laboratory (next ESSL), extreme weather events increase every year in Europe. Many of these extremes have a devastating impact on transport (relocation of goods and people in vehicles) as well as on transport infrastructure (roads, railways, waterways, and transport facilities). Third, according to the conclusions and recommendations from the RAIN project, it is necessary to take into account more significant extremes of weather in the reconstruction of transport facilities than was the case according to technical standards around 1970 (50 years ago). In our research, the worst-case scenarios for transport infrastructure are landslides, snow avalanches, flooding of transport infrastructure, extremely high and extremely low temperatures, storms, intense storms, and forest fires [35].

## 3. Materials and Methods 

**Figure 2:**
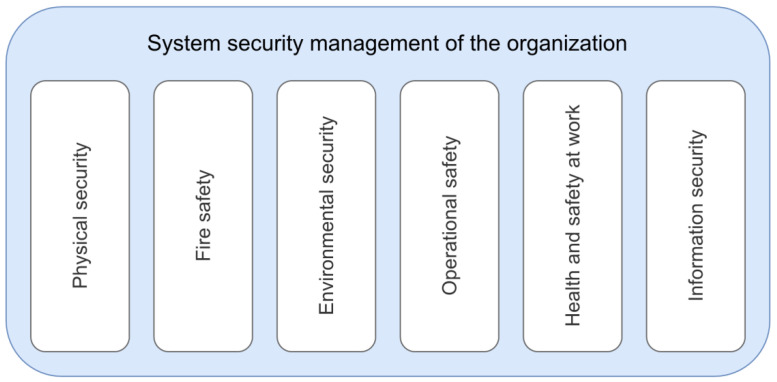
Security pillars of the organization [36].

In our research, standard scientific methods were used, from analysis and comparison to synthesis, from top-down solutions to bottom-up solutions. The basis of all methodological procedures is the definition of the object/system where the risks will be assessed. Probabilities and possible implications for the identified typological scenarios are required as input for all scenarios. Thus, risk assessment can rely on the pillars of system security management of the organization (see Figure 2).

A comprehensive assessment of the safety, security, and resilience of transport infrastructure requires new approaches. Our primary objective should be specifically to reduce the volume of transoceanic traffic in the future. All goods and commodities transported over more than 10,000 km should be subject to a special tax. Moreover, the new scientific department of ecological economics recommends re-evaluating long-distance transport. In the current conditions, there are cases where the raw material obtained on one continent is transported to another continent. The product made of the raw material travels thousands of kilometers to another continent, and if not sold there, it is, in turn, transported to another continent. These cases are possible thanks to a non-comprehensive global solution, wherein some countries do not give attention to the environmental impacts of transport [10]. On the contrary, the security solution requires a comprehensive approach, the security pillars shown in the Figure 2 according to the research results must be evaluated using a comprehensive set of measurable indicators.

Figure 2 shows environmental security as one of the pillars of an organization’s security. Environmental risk assessment is part of a general approach to risk assessment. The current basic framework for risk assessment is ISO 31,000 Risk Management—Guidelines [37]. Various techniques and methodologies for risk assessment are used for different areas of society. As part of the methodology aimed at increasing the security of the organization, it is necessary to link these pillars with the PDCA method (i.e. Plan-Do-Check-Act), which sets out to continuously improve business processes.

**Figure 3:**
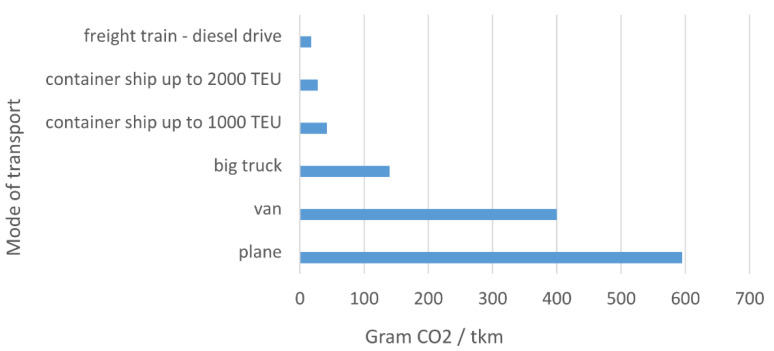
Comparison of CO_2_ emission from the transport of one ton of goods per km [42].

In addressing environmental risk assessment, it is necessary to build on previously conducted research published in these papers and sources focused on air transport [32,35,38,39,40,41] and other modes of transport [33]. A multi-disciplinary and multi-level approach is the basic method for addressing risks. In the risk assessment, it is important to have relevant statistical files for the last period. Another important part of objective risk assessment is the study of best practices in international research. Some innovative approaches are not based on real research tasks but arise as a real need of society. An example of this is the comparison of CO_2_ emission from transport published in Norway as a real answer for the discussion of experts. The real measurements that were taken, gave the results shown in Figure 3. The conversion involved the transport of one ton of cargo over one km and, after the calculation, the real emissions in grams were converted. The lowest value was given by a freight train with a diesel drive. In second place, there is a container ship carrying more than two thousand containers. On the opposite side of the line, was the carriage of one ton of cargo by plane.

For the most part, the aim of future research will be the interconnection of individual subsystems, presented by individual pillars of safety/security and their mutual integration. Future security audits must be based on dozens of indicators for each safety/security pillar. The interconnection and thorough evaluation of the measured values will give a clear answer as to which indicators in which pillar appear the weakest and which we must pay primary attention to. The environmental dimension of the solution must include the entire biodiversity of the environment, starting with microorganisms across plants and animals [7].

The growing number of people on the Earth, the occupation of an ever-increasing area of previously untouched nature for agricultural activity, the growth of urban agglomerations, and the depopulation of rural settlements has long been indeterminate. If the principle of sustainable development of society is correct, it cannot be tested in laboratories, but in natural conditions, thus, it is dysfunctional. In relation to this, it is necessary to perceive every extraordinary event, every deviation from normal, as a challenge for the future in this principle. Research teams must continuously prepare scenarios for even the less probable events. Due to this fact, it is necessary to learn from the crown of the crisis and prepare for similar events [15].

The current crisis caused by COVID-19 shows that even the least likely events can cause gigantic material damage, financial and human loss. Based on these facts, it is necessary to look for new approaches to addressing environmental risk assessment. Routine, template approaches obscure the mind and lead us to a dead end.

## 4. Results

Real research results have been realistically tested by various methods at different levels. One of the possibilities was brainstorming within scientific conferences in combination with a comparison of the results of foreign research projects. The ambition of the authors is to shift knowledge in the field of theory as a theoretical contribution and cooperation with practice as a practical contribution. Another proven method is the preparation of research projects in close cooperation between academia and companies. This trend was applied until 2018. Then, however, there was a turning point, in that currently, projects focused dealing with safety and risks are prepared in cooperation with the state, region, city, municipality with academia, technology companies. Moreover, the fourth group, we invite to cooperate are non-profit stakeholders, associations, and relevant organizations of active people.

This approach brings us significant added value, which lies in a comprehensive understanding of security and risks. All relevant players must be involved in solving these projects. A good example is the current cooperation of regional self-government with the university, companies, state authorities in the region, and interest volunteer associations in the preparation of projects focused on SMART/SAFE city/region.

In this context, the advantage of our faculties is that the authors have been working for a long time on partial methodologies, detailed procedures and solutions that lead to an increase in the level of security. Every year, our security faculties in Zilina and Ostrava discuss dozens of final theses dealing with selected scenarios to prepare the best measures for resolving crisis events.

If we use the division of security areas as presented in Figure 2, one can choose from the reservoirs of partial solutions focused on physical security. These projects have been solved by students in three levels of university studies for more than 10 years. The basic ambition is to prepare security projects designed for all types of facilities, starting with elements of critical infrastructure, across the protection of soft targets to the protection of common facilities according to the requirements of practice.

The second important area where our faculties work closely together and have more than 10 years of results are fire safety projects. These projects are divided into two large groups. Whereas the first one is focused on preventative measures, the second group of projects comprises improving the intervention activities of the fire and rescue corps.

The third area we pay attention to, is the area of occupational safety and health. Primarily, the Faculty of Safety Engineering of the VSB—Technical University of Ostrava has a long tradition of such final theses. This tradition has lasted for more than 20 years. Cooperation in the field of occupational safety and health at the international level also leads to participation in jointly organized conferences and international projects.

The field of environmental safety has markedly a cross-sectional character. Every year, individual final theses are created, the aim of which is to look for methods, processes, and techniques on how to improve environmental protection. The young generation at our faculties is involved in a wide range of youth activities aimed at protecting the environment.

Another important area that has taken on significance within the COVID-19 pandemic is information security. Telework/home office, the use of new communication tools, and the company’s interest in the further development of informatization are the driving force for students to focus their upcoming challenges in the field of cybersecurity in their final theses and projects.

The last presented pillar is technological/technical safety, which is associated with the object/service or real activities. Measures in this area are specified depending on the sector. Within transport, each mode of transport has its own historical development in the field of risk assessment. In the first place, there are space flights, where the required level of safety/acceptable risk has safety indicators set very high. Beside the space flights, attention will also be drawn to the common modes of transport used daily by the population. Next in line is air transport, where previous incidents and air disasters have set safety/security level requirements and safety/security indicators from commonly used modes of transport to the highest level. Safety/risks in air transport are the most important parameter for passengers. As a result of this, in case of any problem before the take-off of the aircraft, each passenger accepts the decision of the captain of the aircraft to postpone the take-off. It is common for passengers who boarded a plane to wait patiently for the captain’s next decision. If they are asked to disembark the aircraft and return to the departure hall, they shall respect such a decision with understanding. In other modes of transport, we will not encounter such an attitude on the part of passengers [12,32,38].

An example of good practice in maritime transport is seaports, which also faces increased control to reduce externalities such as air pollution, noise, and other environmental impacts. Possible sources of emissions in ports include seagoing vessels, port vessels (pilot boats, police boats, pusher tugs), equipment for handling cargo in road transport, semi-trailers, or the trailer sets transporting goods respectively, diesel locomotives, small power plants, industrial, and production centers located in the port.

The emissions from seagoing ships in ports endanger not only the port workers themselves but also the people living near those ports. These emissions cause the population various respiratory diseases such as asthma, lung cancer, and others. For this reason, seaports take various measures to reduce emissions, e.g.,

- use of alternative fuels, or fuels that contain a lower percentage of sulfur;
- the supply of electricity to vessels at berth in ports (in smaller seaports, where there is not enough electricity capacity, vessels generate electricity themselves by burning fuel oil, or diesel);
- improving the exchange of information between vessels and port management centers, which monitor the movement of vessels in ports so that ships can sail at optimum speeds;
- only vessels whose engines meet the technical criteria set out in MARPOL Annex VI may sail in the water area of the port;
- strengthening the control of vessels by the authorities carrying out checks on their technical condition;
- the identification of other areas in the world for the regulation of emissions because of the differences among the countries of the world.

Inland water transport has an exceptionally long history. Risk reduction measures within the technological/technical phase have gradually come with the development of engineering, electronics, energy, and information and communication technologies. At present, however, inland waterway transport lags far behind air transport. In the field of safety, national and international rules and regulations apply. These rules and regulations are accepted, checked, and amended from time to time. Hence, risk assessment in water transport is in its infancy, because some current legal norms and regulations do not accept the current level of knowledge (applies to the countries of central Europe) [43].

Speaking of railway transport, the railway risk assessment was launched within the European Railway Agency by the approval of the Common Safety Methods. A pivotal role of this approach is to find the best practices in other railway companies. The strict separation of the management of railway infrastructure from rail transport has initially led to problems, especially in dealing with the consequences of incidents and railway accidents. The system of renewal of railway infrastructure has for many decades used all the forces and resources of the state railway company for renewal. In addition to this, separating freight and passenger transport from rail infrastructure management has led to complications, prolonging recovery times, and reducing the efficiency of the use of forces and resources. The current situation can be assessed in our countries as follows. Already in the period 1994–1998, the Czech Republic reconstructed a large part of the main transit lines (part of TEN-T) to a speed of 160 km per hour. Currently in the years 2018–2020, it continues in the next phase of reconstruction and modernization of corridor lines. In contrast, Slovakia and Serbia have failed to modernize corridor lines in the last 30 years. The technological progress made by top railway companies in China, Japan, France, and Germany is also an inducement for the countries of central and eastern Europe. The European Railway Agency has defined a role for individual national railway infrastructure administrations and railway undertakings to implement rail risk assessment systems by 2012. Research institutions in the Czech Republic, Slovakia, and Serbia have been intensively involved in this topic. Unfortunately, the transfer of the results of research projects into practice has not taken place yet [19,34,44].

Risk assessment in road transport was addressed in several research projects and the results were published, e.g., in an article by Dvorak et al. [21] and the paper by Fuchs et al. [25]. The development of intelligent transport systems/telematics solutions was started in the Czech Republic, Slovakia, and Serbia around the year 2000. All three countries significantly built the highway network and at the same time implement the results of applied research into the safety of tunnels, which have currently become a good basis for building networks supporting autonomous transport systems [34].

In addition to the area of risk assessment, the writers of the article, along with research teams, attended to the assessment of the vulnerability and resilience of transport and transport infrastructure. OKISD projects [28] were gradually solved, where attention was paid to the foundation of a theoretical framework for the construction of critical infrastructure in transport. The researchers focused mainly on road and rail transport. All workplaces of the Faculty of Security Engineering in Zilina were involved in the solution of the project. Ultimately, the result was more than 100 publications, where the results of the research were presented from different perspectives. A multi-disciplinary and multi-level approach has become the basis of the solution proposed. In the previous phase of the solution, we defined the technical terms that were used in the entire project solution.

The second important contribution was the 7FP RAIN project [18]. Within this project, attention was paid to assessing the risks of extreme weather conditions with priority given to transport infrastructure. As a result of project, vulnerability assessment tools, emphasizing the social vulnerability of society in the event of failures in the energy and transport sectors have been identified.

The most recent project, which brought together the efforts of researchers from Ostrava and Zilina, was the RESILIENCE 2015 project [29]. The project focused on creating a resilience framework for important sectors—energy, transport, and information and communication technologies. Certified methodologies were developed within the project and the results were published in Safety and Security Issues in Technical Infrastructures [34], Dynamic Impact Modeling as a Road Transport Crisis Management Support Tool [45] and in Complex Approach to Assessing Resilience of Critical Infrastructure Elements [46]. The newly developed resilience methodology used a system where resilience consists of adaptability, robustness, and resilience.

**Figure 4:**
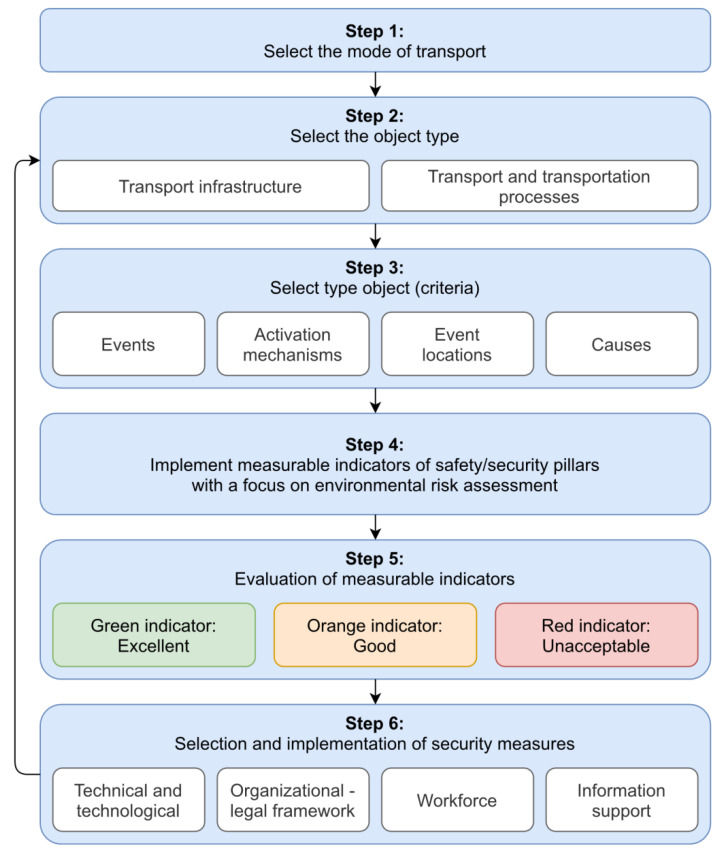
Environmental risk assessment procedure in transport.

Based on the above, it can be stated that the essence of the proposed qualitative approach to environmental risk assessment (QAERA) in transport is the information support of entities in risk management. This approach is determined by 6 pillars of safety and protection of infrastructure elements in the transport sector. More precisely, the environmental risk assessment itself is based on the application of selected safety and security indicators and selected risk assessment criteria in the implementation of environmental change in the global model of thinking in the transport sector. The process of evaluation and use of indicators and criteria is presented in Figure 4.

Step 1: Select the Mode of Transport

The article is devoted to environmental risk assessment in transport and since each mode of transport has certain peculiarities, it is necessary to focus first on one selected mode of transport (air, water, road, or rail).

Step 2: Select the Object Type

After selecting the mode of transport, it is necessary to choose whether the evaluator’s attention will be focused on the transport infrastructure or the transport and transportation processes. When determining reference objects, the division into area (railway stations), line (line sections), or point objects (stop, person, train) is used [46].

The specifics of both types of objects are unambiguous. When assessing threats, the most significant threats to transport infrastructure are, for example, floods and landslides. When assessing threats to transport and transportation processes, the most significant threats include, for example, the intentional act of an employee, the economic crisis or neglecting of work duties.

Step 3: Select Type Object (Criteria)

This step sets out to define the database of type threats (i.e., risk type criteria) of specific reference objects. Events, activation mechanisms, event locations, and causes must be clarified within the database (see Table 1).

After combining the activation mechanism and the source of risk, the following disruptive events arise, for example: malfunction of the security device; immobility of the towing vehicle; illness of staff; panic of the traveling public; panic of railway employees; insolvency of a railway organization; insolvency of debtors of the railway organization; violation of the geometric position of the track; damage to the structure in the body of the track; and fire near the track.

Step 4: Implement Measurable Indicators of Safety/Security Pillars with a Focus on Environmental Risk Assessment

Subsequently, in the fourth step, all safety/security pillars (different viewing angles) must be evaluated. When implementing safety indicators, it is recommended to define 30–50 measurable indicators for each safety/security pillar. As stated above, the paper presents a qualitative approach to environmental risk assessment, which is based on the document of the United Nations [15]:

- Goal 4
  Target 4.7 To ensure that all learners acquire the knowledge and skills needed to promote sustainable development.
- Goal 9
  Target 9.1 To develop quality, reliable, sustainable, and resilient infrastructure, including regional and transborder infrastructure, to support economic development and human well-being, with a focus on affordable and equitable access for all.
- Goal 11
  Target 11.4 To strengthen efforts to protect and safeguard the world’s cultural and natural heritage.
  Target 11.6 By 2030, to reduce the adverse per capita environmental impact of cities, including by paying special attention to air quality and municipal and other waste management.
  Target 11.7 By 2030, to provide universal access to safe, inclusive and accessible, green and public spaces, in particular for women and children, older persons, and persons with disabilities.
  Target 11.a To support positive economic, social, and environmental links between urban, peri-urban, and rural areas by strengthening national and regional development planning.
  Target 11.b By 2020, to substantially increase the number of cities and human settlements adopting and implementing integrated policies and plans towards inclusion, resource efficiency, mitigation and adaptation to climate change, resilience to disasters, and develop and implement, in line with the Sendai Framework for Disaster Risk Reduction 2015–2030, holistic disaster risk management at all levels.
- Goal 13
  Target 13.1 To strengthen resilience and adaptive capacity to climate-related hazards and natural disasters in all countries.
  Target 13.2 To integrate climate change measures into national policies, strategies, and planning.
  Target 13.3 To improve education, awareness-raising, and human and institutional capacity on climate change mitigation, adaptation, impact reduction, and early warning.

However, the writer’ research was aimed at defining the required number of environmental safety indicators. For this reason, 38 indicators were defined for the real information system. In the following, we introduce proposals concerning some measurable indicators. The evaluation of measurable indicators using the procedure described below will give a clear conclusion about the level of safety of the reference object against a specific source of risk with a proposal for specific measures to reduce it. Examples of some indicators are presented in Table 2, Table 3 and Table 4.

The issue of environmental safety/security indicators is closely related to technical standards, laws, and regulations. Without deep experience in the field of environmental protection, it is not possible to set indicators that will objectively measure environmental security.

Step 5: Evaluation of Measurable Indicators

The same way as the color displays the resulting values in the traffic light (green, orange, and red), it is possible to determine the current level of safety. Briefly, if the values are, for example, on the orange scale, it is appropriate to monitor the indicator. If it is found out that an indicator was included in the green zone during testing, then it is not necessary to conduct safety measures. When entering the orange zone, it is necessary to increase vigilance, limit the speed of traffic, and, if necessary, choose appropriate safety measures to increase safety and security. If the indicator is included in the red zone, it is necessary to stop the transport process and take appropriate safety/security measures immediately.

Step 6: Selection and Implementation of Security Measures

The sixth step is a set of measures based on practical experience. Sets of proposed measures are created for the areas:

- technical and technological,
- organizational—legal framework,
- workforce,
- information support.

The technical and technological area is very important, as there is a system of planned inspection and maintenance. Namely, rail transport has a proven system of real technical and technological measures for years to guarantee track safety. Organizational measures are based on four sources. The first source is legal acts—laws, and decrees at the national level, the second is regulations and recommendations at the international level, the third is technical standards, and the fourth is the internal regulations of the infrastructure manager and transport companies. All listed resources are updated at regular intervals. In the area of manpower, there exist measures for the selection, training, and continuous training of employees. That is why railway companies have a long-established training system. For the most part, there are significant changes in the area of information support. Historical security and notification devices have been gradually replaced by high-quality information and communication technology. A current problem in this area is the provision of information security, which is carried out on an ongoing basis in all railway undertakings. Measures in this area focus on people, hardware, software, and data protection (GDPR, sensitive information, and know-how).

Practical application of the decision-making process qualitative approach to environmental risk assessment (QAERA) provides transport. As part of the verification process proposed by QAERA, several scenarios had to be prepared where the whole approach would be tested. The real events from the recent past played decisive role in the selection of scenarios. The first scenario was set by prolonged heavy rainfall which caused large-scale floods. The second scenario for landslides was made up after intense flash floods. The third scenario focused on the intentional act of a railway company employee. Each of these scenarios has a significant environmental impact. Each is relatively likely with a possible high impact on rail transport.

## 5. Discussion

The qualitative approach to environmental risk assessment in transport is based on long-term research, which relies on the historical basis of our research organizations. The second key part is the company’s interest in environmental safety as one of the priorities [7,8,15,25,30,47]. If we compare different sectors of the national economy, we will find out that the issue of environmental risk assessment is addressed at different levels. Good practice is mostly elaborated in the field of chemical industry and nuclear facilities. In transport, good practice is exemplified in Polishchuk et al. [33] in a fuzzy model of risk assessment for environmental issues. In rail transport, the attention is drawn to the implementation of Common Safety Methods [48], also with reference to examples of good practice.

The current approaches, in which the risk assessment is narrowed down to a defined system, the identification of threats, the analysis of risks, and the design of measures for the selected risk for a specific object/service, are, as mentioned, very narrowed down. The innovativeness of our solution rests on the interconnection of several approaches from vulnerability assessment, alternatively ranging from the assessment of resilience to security indicators as part of the safety/security pillars. The complexity of the solution should take into account all interests—global, national, regional, corporate, and individual. Creating an intelligent/SMART and comprehensive environmental risk assessment solution requires a real scientific basis, long-term experience, and a multidimensional perspective solution [35,49]. Previously implemented projects focused on static evaluation, however, the current trend is focused on the dynamic dimension of the solution [45]. The writers of the article are aware of the need to create a theoretical framework, followed by a methodological procedure and a detailed algorithm of selected scenarios, which will be the basis for the IT solution. In the past, it was not possible to create a comprehensive information system that would provide real-time technology using of the technologies, e.g., the internet of things, big data, and cloud, to gain all the necessary data for decision-making by top managers responsible for environmental risk assessment.

The interconnection of computer science, environmental, and security sciences is a challenging us at present. A multidisciplinary solution, using the knowledge of all relevant disciplines, is a key tool for a comprehensive qualitative approach to environmental risk assessment. Transport, as one of the economic sectors, requires further innovation and research. The real possibility is the continuous improvement of technical and technological processes for individual transport subsystems: transport technology, transport infrastructure, control and information systems in transport, and education and training of transport workers. This original method was intended for the regional and national levels. It does not only address global issues that require a different understanding of current needs and risk management. Moreover, when seeking a global solution, a multi-criteria assessment, which appropriately favors environmental aspects, must be used. Thousands of unused trucks cannot drive on the roads together with the thousands of overloaded trucks. Furthermore, environmentally friendly modes of transport such as water and rail cannot have smaller volumes of transported goods from year to year (situation in central Europe). Furthermore, it is not reasonable to increase the volume of goods transported in maritime transport every year, without full utilization of domestic resources in individual countries.

It is necessary to gradually fulfil the global model of sustainable development [15] step by step so that each activity carried out, reflects the protection of the environment. Promoting globally defined 17 goals and 197 targets are one of the right paths to global environmental protection.

Current quantitative approaches to risk assessment are based on long-term records and available risk data. However, individual countries in this area are creating a database only gradually. Therefore, at this stage of development, the attention of researchers was focused on qualitative evaluation, which also uses other data and thus does not need an extensive database. Thus, the result of this assessment is the identification of risk areas that can be evaluated in the next step using quantitative methods.

## 6. Conclusions

The paper aimed to present the ideas, reflections, projects, and publications of writers who have long been engaged in research in the fields of transport, safety, and the environment. We are aware of environmental impacts possibly affecting the functioning of transport and the operability of transport infrastructure. With regard to this, they certainly affect people’s health and lives. Every year, millions of people around the world are exposed to new extremes in the weather. Due to them, there is a movement of nations, whose inhabitants are forced to leave their homes and search for new places to live. Globally, the 50 percent limit of urban settlement has been exceeded, thus with more than half of mankind currently living in cities and conurbations with an absolute dependence on the functioning of critical infrastructures.

Research on dynamic resilience has shown us the complexity of current systems, their interdependence and considerable vulnerability. The more complicated and intelligent systems that control the sources of human needs is, the greater the possible impact on people’s lives and the environment.

Safety/security research leads us to ever new challenges how to increase the level of resilience, how to improve the protection of people, companies, institutions, municipalities, cities, regions, and, in general, of the globe. In our research, we have repeatedly encountered the limits of what is feasible, as we solved clearly defined problems and, interestingly, new and new questions have emerged as solutions.

The current limitations of the research play pivotal role, as they need to be based mainly on the multi-specialization of the solution. Creating broad teams of experts in practice and connecting researchers at the international level can bring about a shift in risk assessment in transport. Another limitation is the absence of legal acts that individual countries should issue in order to reduce environmental risks. Last but not least, there is the stereotype that people are used to pollute the environment and do not consider it a major problem. Awareness, education, and training must be coordinated and clear standards for environmental education and training must be defined in the future.

The field of applied research and innovation must be directed to the creation of new approaches, new methodologies, and new techniques in the field of environmental risk in transport. A comprehensive approach and timely identification of sources of risk is key. The writers demonstrated a new approach applicable in environmental risk assessment in transport by proposing safety pillars and a set of indicators.

The issue of environmental risk assessment in the countries of central and eastern Europe is addressed only in research organizations. It cannot be shifted into a real transport policy. It solves other seemingly more important problems—the moral obsolescence of transport infrastructure, non-coordination of individual modes of transport, corrupt behavior, and the absence of SMART solutions/technologies. Research results are rarely used as a basis for government negotiations at national and regional levels. In the area of transport policy, there is a lack of coordination of solutions at national and regional level. To sum up, the proposed approach provides six basic pillars of safety and dozens of indicators that could be part of the transport agenda at local, regional, and national level.

Based on the above, safety/security research requires teamwork, preferably with the representation of experts from different disciplines, because the perception of security is very individual. If we want to bring truly comprehensive solutions, it is necessary to start from all relevant research areas and look for synergistic solutions that can bring a new quality of security to society.

## Figures and Tables

**Table 1:** Criteria determining the type of risk.

Areas	Criteria
Event naming:	1. Exclusion of traffic—the threat may manifest itself in that it is not possible to operate train transport. 2. Change of the schedule—the threat may manifest itself in such a way that it is possible to operate traffic, but to a limited extent. 3. Technology change—threats can manifest themselves as an impact on the work system. 4. Change in the scope of services—the threat may manifest itself in such a way that the infrastructure is not disrupted, but there has been a change in services.
Activation mechanisms:	1. Mass infection. 2. Assault on operating personnel. 3. Device malfunction. 4. Illness of staff. 5. Panic. 6. Fire. 7. Landslide. 8. Flooding of the runway.
Event locations:	1. Train—point object. 2. Track section—line object. 3. Station—area object. 4. Surroundings of the track—area object.
Causes of events:	1. Unintentional errors of transport participants. 2. Failure of supplies necessary for operation. 3. Problems in state administration. 4. Extreme natural phenomena and weather. 5. Technical failure. 6. Crime.

**Table 2:** Indicator—Amount of carbon dioxide (CO_2_) released into the air.

Value	Verbal Expression	Description	Units/Percentages
1	excellent	Amount of CO_2_ emissions produced from biomass used as fuel, including CO_2_ emissions that were not caused by the use of biomass in quantities ranging from 0 to 110 Mg per year.	from 0 to 110 Mg per year
2	good	Amount of CO_2_ emissions produced from biomass used as fuel, including CO_2_ emissions that were not caused by the use of biomass in the range of 110 to 162 Mg per year.	from 110 to 162 Mg per year
3	unacceptable	Amount of CO_2_ emissions produced from biomass used as fuel, including CO_2_ emissions that were not caused by the use of biomass in quantities exceeding 162 Mg per year.	more than 162 Mg per year

Comment: The indicator presents the measurable production of CO_2_ emissions from biomass.

**Table 3:** Indicator—Amount of investments aimed at environmental protection.

Value	Verbal Expression	Description	Units/Percentages
1	excellent	The railway infrastructure manager spends € 100,000 or more per year on environmental protection.	100,000 euros and more
2	good	The railway infrastructure manager spends between €30,000 and €99,999 per year on environmental protection.	30,000 to 99,999 euros
3	unacceptable	The railway infrastructure manager spends less than €30,000 a year on environmental protection.	less than 30,000 euros

Comment: The indicator presents the amount of investments of the railway infrastructure manager intended for environmental protection.

**Table 4:** Indicator—Status of equipment for measuring meteorological data.

Value	Verbal Expression	Description	Units/Percentages
1	excellent	Checks of the functionality of measuring equipment are performed at regular intervals.	90%
2	good	Checks of the functionality of measuring equipment are performed at irregular intervals.	70%
3	unacceptable	The state of functionality of measuring equipment is outdated, the results of measured values are inadequate.	30%

Comment: The indicator presents the status of the meteorological equipment of the infrastructure manager used as a database for telematics solutions in transport.
